# Effects of kinesio taping on lower limb biomechanical characteristics during dynamic postural control tasks in individuals with chronic ankle instability

**DOI:** 10.1371/journal.pone.0317357

**Published:** 2025-01-10

**Authors:** Tao Yuan, Haixia Li, Guanglan Wang

**Affiliations:** 1 Wuhan Children’s Hospital (Wuhan Maternal and Child Healthcare Hospital), Tongji Medical College, Huazhong University of Science & Technology, Wuhan, Hubei Province, China; 2 Key Laboratory of Sports Engineering of General Administration of Sport of China, Wuhan Sports University, Wuhan, Hubei Province, China; 3 School of Sports Medicine, Wuhan Sports University, Wuhan, Hubei Province, China; University of Tehran, ISLAMIC REPUBLIC OF IRAN

## Abstract

**Purpose:**

Previous studies have demonstrated significant biomechanical differences between individuals with chronic ankle instability (CAI) and healthy controls during the Y-balance test. This study aimed to examine the effects of kinesio taping (KT) on lower limb biomechanical characteristics during the Y-balance anterior reach task in individuals with CAI.

**Methods:**

A total of 30 participants were recruited, comprising 15 individuals with CAI and 15 healthy controls. All participants were randomly assigned three taping conditions: no taping (NT), placebo taping (PT), and KT, followed by the Y-balance anterior reach task. Each condition was separated by one-week intervals. Kinematic and kinetic data of the lower limbs during the movement phase were collected using the Vicon motion capture system (Vicon, T40, 200 Hz) and two Kistler force platforms (Kistler, 1000 Hz).

**Results:**

KT significantly improved the Y-balance anterior reach distance (*P = 0*.*003*) and peak ankle eversion angle (*P* = 0.019) compared to NT. Additionally, KT resulted in increased peak knee flexion angle (*P* = 0.002, *P* = 0.011) and peak ankle dorsiflexion angle (*P* <0.001, *P* = 0.005) relative to both NT and PT. KT also significantly reduced mediolateral center of pressure (COP) displacement (*P* = 0.001) and average velocity of mediolateral COP displacement (*P* = 0.033) in comparison to NT. Furthermore, KT decreased mediolateral center of gravity displacement (*P* = 0.002, *P* = 0.003) relative to both NT and PT.

**Conclusion:**

KT significantly improved abnormal ankle posture by promoting greater ankle dorsiflexion and eversion angles. Additionally, KT reduced mediolateral COP displacement and average velocity to improve postural stability. These changes may contribute to reduced risk of ankle sprains. Therefore, KT may serve as an effective tool for managing recurrent ankle sprains in individuals with CAI.

## 1. Introduction

Ankle sprains are among the most common musculoskeletal injuries in sports [1]. After recovering from acute trauma, up to 40% of individuals with ankle sprains may develop chronic ankle instability (CAI) [2]. Those with CAI frequently experience episodes of the ankle “giving way” and are susceptible to recurrent sprains [3]. These recurrent injuries exacerbate the instability, creating a vicious cycle of sprain-instability-reinjury [4]. Without proper intervention, this cycle can persist indefinitely, leading to a range of adverse health consequences. Research has shown that individuals with CAI face a higher risk of post-traumatic ankle osteoarthritis and reduced physical activity. Additionally, they experience a decline in health-related quality of life [5, 6]. A key factor in recurrent sprains among individuals with CAI is impaired postural stability [7]. Ankle sprains can damage peripheral receptors, including muscle spindles, Golgi tendon organs, Ruffini corpuscles, and Pacinian corpuscles. This damage hinders the ability of the central nervous system to accurately make correct judgments about body changes or external interference, resulting in adaptive changes or reorganization. Ultimately, this leads to reduced or inaccurate motor output, contributing to decreased postural stability [8].

Kinesio taping (KT) is a therapeutic method that approximates the thickness of the skin and has been shown to effectively improve postural stability in individuals with CAI [9, 10]. By stimulating mechanoreceptors in the skin, KT activates proprioceptors, increasing sensory input signals, thereby promoting the central nervous system to establish new sensory pathways [11, 12]. Additionally, high-elasticity KT not only provides superior comfort but also avoids limiting range of motion in the ankle compared to athletic taping, soft braces, and orthotics [13–16]. This helps prevent a stiff landing pattern in the lower extremities, thereby reducing the risk of sports injuries [17]. The Y-balance test is a widely used measure of postural stability. Prospective cohort studies have shown that a reduced anterior reach distance in this test is a significant risk factor for ankle sprains [18–20]. While previous studies have examined the effects of KT on balance function in Y-balance tasks among individuals with CAI, few have focused on the biomechanical factors underlying these effects [21–23]. However, existing research suggests that biomechanical parameters, such as knee flexion and ankle dorsiflexion angles, are significantly positively correlated with Y-balance anterior reach distance [24, 25]. This indicates that biomechanical parameters may influence performance on the Y-balance test. Furthermore, biomechanical testing, as a highly precise tool, can directly identify risk factors associated with sports injuries. Therefore, evaluating the effects of KT on biomechanical characteristics during Y-balance tasks can assist researchers and clinicians in developing rehabilitation plans targeted at ankle sprain prevention.

Sarvestan et al. [26] found that KT reduced mediolateral postural sway velocity during single-leg stance in individuals with CAI, suggesting that KT can enhance static stability. Many complex movements in daily life involve dynamic balance, which can more effectively reveal postural stability deficits in individuals with CAI [27]. However, there are no studies have evaluated the effects of KT on biomechanical characteristics during dynamic postural stability tasks. This study aimed to compare the differences in lower limb biomechanical characteristics between individuals with CAI and healthy controls during the Y-balance anterior reach task across three conditions: no taping (NT), placebo taping (PT), and KT. That can provide information and reference for developing ankle sprain management strategies for individuals with CAI. We hypothesized that KT would increase peak dorsiflexion and eversion angles while reducing mediolateral COP displacement and average velocity.

## 2. Methods

### 2.1 Participants

A total of 30 participants were recruited for this study—15 with chronic ankle instability (CAI) and 15 healthy controls. All participants were recruited from universities in the Wuhan region between November 5, 2023, and November 20, 2023. Out of 200 eligible individuals screened, 100 expressed willingness to participate, resulting in an agreement rate of 50%. This recruitment represents 15% of the source population. Data were collected at the Key Laboratory of Sports Engineering of General Administration of Sport of China, Wuhan Sports University. All participants were informed of the experimental procedures and provided written informed consent forms before data collection. This study was approved by the medical ethics committee of Wuhan Sports University(No. 2023083). The inclusion criteria for participants with CAI were: (1) a history of at least one severe lateral ankle sprain occurring at least 12 months prior to enrollment; (2) the most recent ankle sprain occurred three months ago; (3) participants should exercise regularly, more than three times per week, with each session lasting over 30 min; (4) a score of ≤24 on the Cumberland ankle instability tool (CAIT) [28]; and (5) The affected ankle has experienced “giving-way” or instability at least twice in the past six months [29]. For healthy control group, inclusion criteria included (1) a CAIT score of ≥28 [30]; and (2) no history of lateral ankle sprains or other ankle injuries. The exclusion criteria were as follows: (1) musculoskeletal injuries in the lower limbs within the past three months; (2) balance disorders; and (3) other acute medical conditions.

### 2.2 Taping methods

The testing leg of each participant was taped by a trained professional using 5 cm × 5 m Kindmax tape. The KT protocol and tension were adapted from Wang et al. [31]. This taping method has been proven to reduce ankle plantarflexion and inversion angles, thereby enhancing ankle joint stability [32]. Previous studies have typically used 50% tension to improve muscle function [33, 34]. The formula was as follows:

$$Taping\;cutting\;length=(\frac{actual\;length-8\;cm}{1.5}+8\;cm)\times 1.1$$

**Fig 1:**
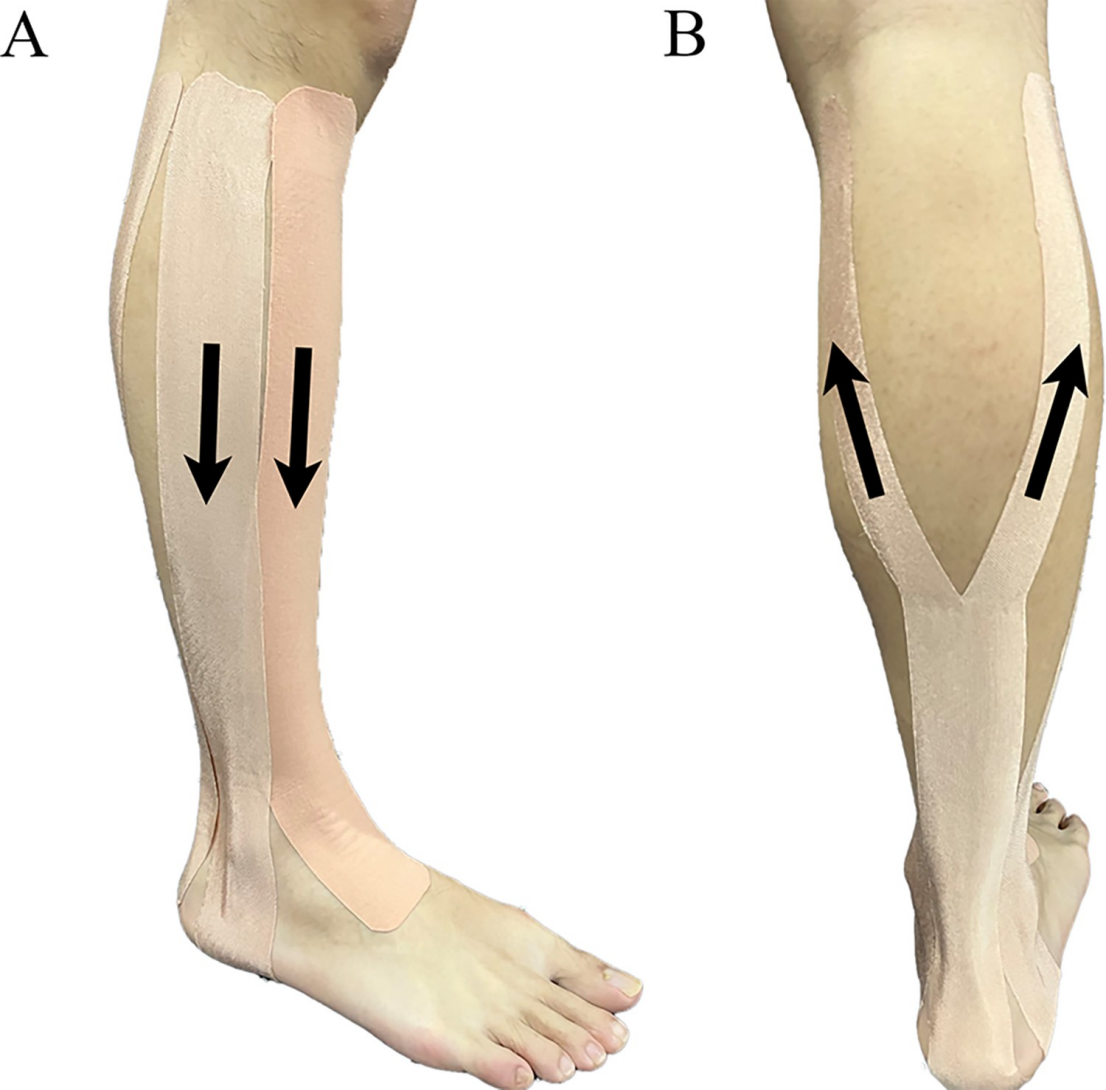
Taping methods under the KT condition. (A) Tibialis anterior (right side) and peroneus longus (left side), (B) Gastrocnemius; Black arrows indicate the taping direction.

The taping procedures were as follows: (1) Tibialis anterior: An I-shaped strip of tape was applied from the tibial tuberosity to the front of the dorsum of the foot, covering the muscle belly of the tibialis anterior. (2) Peroneus longus: Another I-shaped strip was applied from the fibular head to just above the medial malleolus, covering the muscle belly of the peroneus longus. (3) Gastrocnemius: A Y-shaped tape was applied starting at the sole of the foot and extending to the medial and lateral epicondyles of the femur, covering the muscle belly of the gastrocnemius (Fig 1). For PT, the same taping methods were used as in the KT condition, but without any applied tension. In the NT condition, no tape was applied. All participants underwent three interventions in a randomized sequence with a 1-w interval between each condition. Both the researchers and the participants were blinded to the randomization order.

### 2.3 Experimental data collection

**Fig 2:**
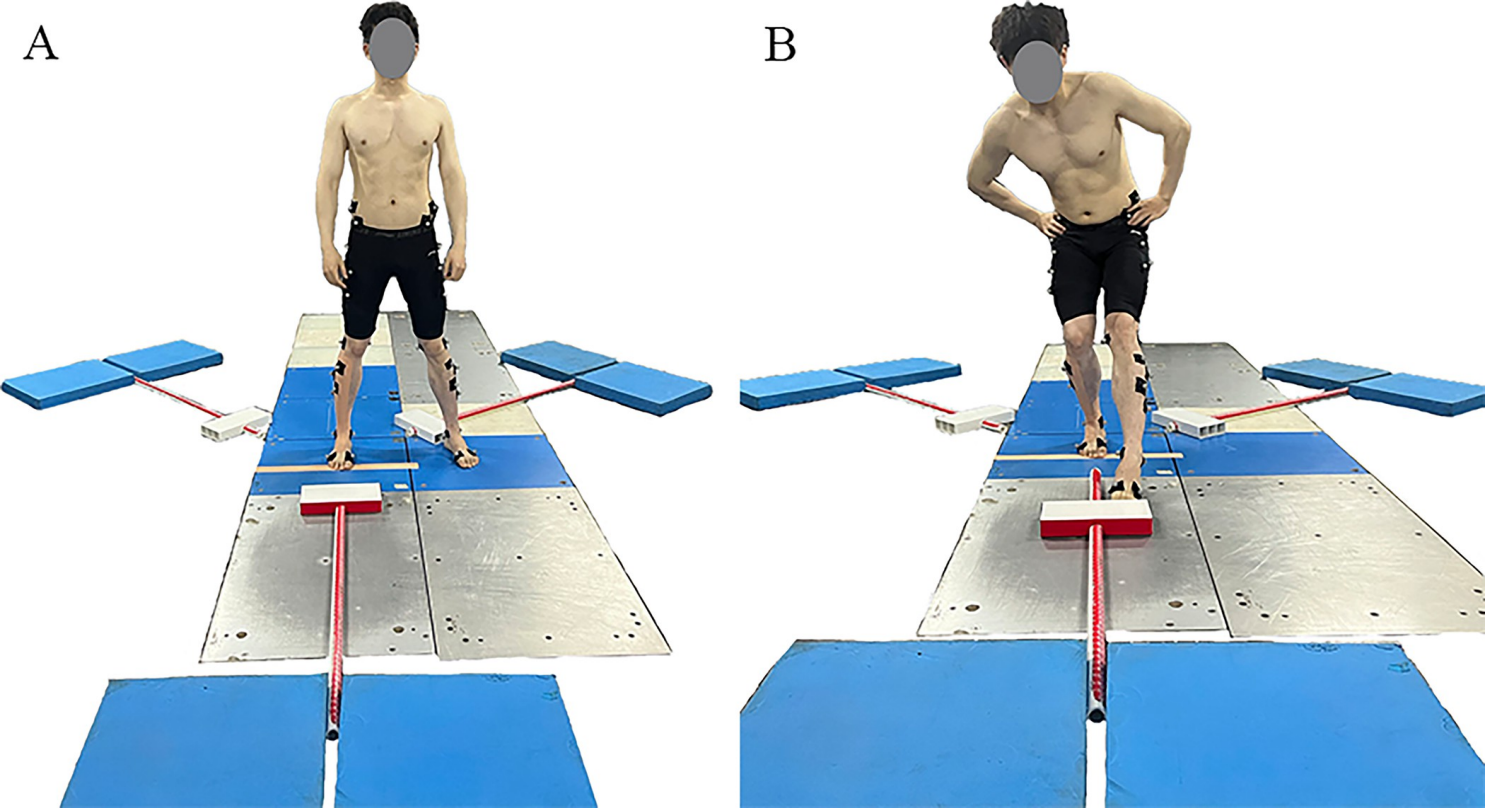
Schematic of the Y-balance anterior task. (A) Starting position, (B) Forward movement.

Participants changed into tight-fitting experimental clothing and performed a 10-minute warm-up on treadmill at a speed of 6.5 km/h. After the warm-up, KT and marker points were applied to the lower limbs of participants. The lower limb model from Chen et al. [35] was employed, which is effective in tracking the motion trajectories of both the pelvis and lower limbs. Once the markers were applied, the participants performed the Y-balance task. For the test, participants stood barefoot on their testing leg at the center of the force platform, with the big toe aligned with a designated white line. The non-testing leg either rested on the ground or on another force platform. Both hands were placed on the hips, and participants were instructed to focus their gaze forward. At the start of the test, participants maintained a single-leg stance and sequentially reached the platform in three directions: anterior, posteromedial, and posterolateral directions (Fig 2). If participants removed their hands from their hips, moved their foot, or lost balance, this data was discarded, and new data were collected until three successful attempts were recorded. A 60-second rest period was given between each trial. All participants received training on the experimental movements upon enrollment and completed three practice trials before each test to minimize learning effects. For participants with CAI, the testing leg was the affected side, while for healthy controls, it was the dominant leg. The dominant leg was the preferred leg for kicking actions [36]. Kinematic and kinetic data were collected simultaneously using a nine-camera infrared high-speed motion capture system (Vicon, Nexus, T40, sampling at 200 Hz) and 3D force platforms (Kistler, sampling at 1000 Hz). Our study focused exclusively on analyzing the anterior reach direction. This focus is based on previous research indicating that neuromuscular deficits in the anterior direction are significantly associated with an increased risk of ankle sprains [20].

### 2.4 Experimental data processing

Data were analyzed using Visual 3D (C-Motion, Germantown, Maryland, USA). Measurements included anterior reach distance, kinematics, and kinetics of the testing leg during the movement phase of the Y-balance anterior reach task. Kinematic data encompassed peak hip, knee, and ankle joint angles, while kinetic data included center of pressure (COP) displacement and average velocity, center of gravity (COG) displacements and average velocity, as well as peak hip, knee, and ankle joint moments. The movement phase was defined as the period from the initiation to the task until the point of peak knee flexion. Anterior reach distance was normalized according to the leg length of each participant. Joint angles for hip, knee, and ankle during the movement phase were computed based on Cardan X–Y–Z rotation sequence, and the maximum value was extracted. COP and COG displacements were calculated as the differences between the maximum and minimum offsets within a given plane during the movement phase (the total length of the COP and COG lines). The average velocity of both COP and COG displacements was determined by dividing the displacement distance by the duration of the movement phase. Hip, knee, and ankle joint moments during the movement phase were calculated using inverse dynamics of the Newton-Euler method and normalized to body weight. The maximum values were selected. The data used for statistical analysis were the mean of three experimental measurements.

### 2.5 Statistical analysis

Statistical analysis was conducted using IBM SPSS Statistics for Windows (version 26.0; IBM Corp., Armonk, NY, USA), with the significance level set at *P* <0.05. Categorical demographic variables between groups were compared using Fisher’s exact test, while continuous variables were analyzed with independent t-tests. A two-way mixed ANOVA was used to assess the main and interaction effects of taping and group on outcome measures. If a significant interaction effect was found, a simple effects analysis was conducted; if not, main effects were analyzed. The normality of the data was assessed using the Shapiro-Wilk test, and post-hoc comparisons were made using Bonferroni correction.

## 3. Results

### 3.1 Demographic characteristics

This study included a total of 15 individuals with CAI, with an average age of 19.93 ± 1.83 years, a height of 172.40 ± 6.29 cm, and a weight of 62.47 ± 11.33 kg. Additionally, 15 healthy controls were included, with an average age of 21.20 ± 1.66 years, a height of 171.87 ± 7.04 cm, and a weight of 60.80 ± 10.69 kg. A significant difference in CAIT scores was observed between the two groups (*P* <0.05), while no other demographic characteristics showed statistically significant differences (*P* >0.05). There were no drop-outs; all 30 participants successfully completed the experiment and were included in the analysis (Table 1).

**Table 1:** Demographic characteristics of the two groups.

	CAI (n = 15)	Healthy(n = 15)	P
Sex	Male: 7, Female: 8	Male: 5, Female: 10	0.462
Age (Years)	19.93±1.83	21.20±1.66	0.057
Height (cm)	172.40±6.29	171.87±7.04	0.828
Mass (kg)	62.47±11.33	60.80±10.69	0.682
Testing leg	Left: 3, Right: 12	Left: 0, Right: 15	0.224
CAIT scores	16.53±4.73	30	<0.001

CAI, chronic ankle instability; CAIT, Cumberland Ankle Instability Tool

### 3.2 Y-balance anterior reach distance and kinematic characteristics

There was a significant main effect of the taping condition on Y-balance anterior reach distance. Post-hoc comparisons revealed that, in both groups, Y-balance anterior reach distance was significantly greater under the KT condition compared to the NT condition (*P* = 0.003).

Regarding peak knee flexion angle, a significant interaction effect was found between the taping conditions and participant groups. Individuals with CAI exhibited a significantly increased peak knee flexion angle under the KT condition compared to both the NT and PT conditions (*P* = 0.002, *P* = 0.011). Additionally, peak knee flexion angle in individuals with CAI was significantly lower than that of healthy controls across all three taping conditions (*P* = 0.001, *P* = 0.002, and *P* = 0.035).

There was a significant main effect of the taping condition on peak ankle dorsiflexion angle. Post-hoc comparisons indicated that, in both groups, peak ankle dorsiflexion angle was significantly greater under the KT condition compared to both the NT and PT conditions (*P* <0.001, *P* = 0.005).

Similarly, there was a significant main effect of the taping condition on peak ankle eversion angle. Post-hoc comparisons revealed that, in both groups, peak ankle eversion angle was significantly increased under the KT condition compared to the NT condition (*P* = 0.019; Table 2).

**Table 2:** Y-balance anterior reach distance and kinematic characteristics.

Indicators	CAI			Healthy			Taping	Group	Taping×Group
	NT	PT	KT	NT	PT	KT			
Y-balance anterior reach distance (cm)	66.74± 5.85	66.38± 6.34	68.12± 5.98	67.55± 2.64	67.64± 2.84	68.75± 2.66	**0.003** ^*^	0.737	0.757
Peak hip Flexion angle(°)	33.64± 9.82	33.63± 8.28	34.99± 7.91	30.50± 7.68	32.04± 6.72	32.30± 7.03	0.093	0.383	0.535
Peak knee flexion angle (°)	-69.22± 12.05	-71.95± 10.42	-76.04±10.96	-84.54± 10.21	-84.74± 9.94	-85.60± 12.66	**0.004** ^*^	**0.003** ^*^	**0.048** ^*^
Peak ankle dorsiflexion angle (°)	12.89± 4.26	13.93± 4.98	16.94± 3.75	15.60± 5.91	15.98± 6.21	16.83± 5.43	**<0.001** ^*^	0.382	0.065
peak hip adduction angle (°)	9.00± 4.98	10.75± 5.65	11.59± 4.28	9.88± 7.15	8.58± 6.48	8.61± 6.27	0.793	0.441	0.126
Peak knee valgus angle (°)	-3.37± 4.40	-2.56± 3.29	-2.36± 3.39	-4.31± 2.85	-5.08± 3.03	-4.71± 2.48	0.688	0.095	0.094
Peak ankle eversion angle (°)	-5.57± 3.86	-7.07± 4.72	-7.64± 5.05	-6.98± 2.96	-6.85± 2.82	-7.75± 3.13	**0.01** ^*^	0.742	0.165

CAI, chronic ankle instability; NT, no taping; PT, placebo taping; KT, kinesio taping; COP, center of pressure; COG, center of gravity. *indicates a significant difference (P<0.05)

### 3.3 Kinetic characteristics

There were significant main effects of taping and group on mediolateral COP displacement. Post-hoc comparisons revealed that, in both groups, mediolateral COP displacement significantly reduced under the KT condition compared to the NT condition (*P* = 0.001). Additionally, individuals with CAI demonstrated greater mediolateral COP displacement than healthy controls under all three taping conditions (*P* = 0.045).

A significant main effect of the taping condition was observed on average velocity of mediolateral COP displacement. Post-hoc comparisons revealed that, in both groups, average velocity of mediolateral COP displacement was significantly decreased under the KT condition compared to the NT condition (*P* = 0.033).

A significant main effect of the taping condition was found on mediolateral COG displacement. Post-hoc comparisons revealed that, in both groups, mediolateral COG displacement was significantly reduced under the KT condition compared to both the NT (*P* = 0.002) and PT conditions (*P* = 0.003; Table 3).

**Table 3:** Kinetic characteristics.

Indicators	CAI			Healthy			Taping	Group	Taping×Group
	NT	PT	KT	NT	PT	KT			
Anterioposterior COP displacement (mm)	4.33± 0.91	4.10± 0.87	4.03± 1.00	3.66± 0.74	3.59± 0.94	3.64± 0.66	0.356	0.071	0.505
Mediolateral COP displacement (mm)	10.47± 2.80	9.93± 2.37	8.76± 1.90	8.71± 1.40	8.57± 1.90	7.94± 1.65	**0.003** ^*^	**0.045** ^*^	0.422
Average velocity of anterioposterior COP displacements (mm/s)	0.93± 0.32	1.03± 0.29	0.96± 0.24	0.98± 0.24	0.91± 0.20	0.86± 0.22	0.443	0.472	0.174
Average velocity of mediolateral COP displacement (mm/s)	2.47± 0.95	2.62± 1.33	1.97± 0.79	2.23± 0.38	2.23± 0.73	2.13± 0.67	**0.013** ^*^	0.579	0.102
Anterioposterior COG displacement (mm)	19.54± 2.51	17.70± 3.71	18.48± 3.27	18.13± 2.29	16.50± 3.43	17.01± 3.31	0.065	0.089	0.98
Mediolateral COG displacement (mm)	14.01± 4.20	13.56± 4.00	11.24± 3.30	13.30± 2.18	13.18± 2.12	11.95± 2.11	**<0.001** ^*^	0.899	0.3
Average velocity of anterioposterior COG displacements (mm/s)	4.00± 1.47	4.12± 0.99	4.20± 1.27	3.10± 0.79	3.66± 0.81	3.61± 0.71	0.059	0.061	0.397
Average velocity of mediolateral COG displacement (mm/s)	3.34± 1.87	3.05± 1.30	2.97± 1.39	2.86± 1.00	2.96± 0.99	2.75± 0.46	0.432	0.513	0.573
Peak hip flexion moment (Nm/kg)	1.15± 0.46	1.13± 0.31	1.15± 0.34	0.90± 0.37	1.09± 0.42	1.11± 0.43	0.255	0.362	0.235
Peak knee flexion moment (Nm/kg)	-1.20± 0.28	-1.15± 0.35	-1.16± 0.26	-1.21± 0.32	-1.52± 0.61	-1.25± 0.29	0.256	0.1	0.104
Peak ankle dorsiflexion moment (Nm/kg)	1.06± 0.15	1.07± 0.16	1.09± 0.13	1.05± 0.22	1.00± 0.19	1.02± 0.17	0.775	0.354	0.528
Peak hip adduction moment (Nm/kg)	0.22± 0.12	0.25± 0.16	0.18± 0.09	0.24± 0.13	0.23± 0.10	0.26± 0.11	0.748	0.408	0.222
Peak knee valgus moment (Nm/kg)	-0.34± 0.21	-0.35± 0.17	-0.31± 0.23	-0.44± 0.23	-0.45± 0.27	-0.46± 0.30	0.956	0.063	0.889
Peak ankle eversion moment (Nm/kg)	-0.16± 0.12	-0.16± 0.11	-0.20± 0.14	-0.24± 0.11	-0.23± 0.09	-0.25± 0.09	0.112	0.079	0.856

CAI, chronic ankle instability; NT, no taping; PT, placebo taping; KT, kinesio taping; COP, center of pressure; COG, center of gravity; N, Newton. *indicates a significant difference (P<0.05).

## 4. Discussion

This study employed the Y-balance anterior reach task to investigate the effects of three different taping conditions on lower limb biomechanical characteristics in individuals with CAI and healthy controls. The results demonstrated that KT significantly enhanced Y-balance anterior reach distance, as well as peak knee flexion, peak ankle dorsiflexion, and eversion angles in individuals with CAI. Furthermore, KT reduced mediolateral COP displacement and average velocity of mediolateral COP displacement, along with mediolateral COG displacement. These findings suggests that KT can improve performance in dynamic balance tasks and positively influences lower limb biomechanics related to ankle sprains. Consequently, KT may serve as an effective tool for managing recurrent ankle sprains.

### 4.1 Y-balance anterior reach distance and kinematic characteristics

Postural stability is a fundamental element of body movement control and coordination, playing a crucial role in both athletic performance and injury prevention [37, 38]. Dynamic postural stability, in particular, refers to the ability of the body to maintain stability during disturbances or active movements [39]. The findings of this study indicate that KT significantly improved Y-balance anterior reach distance compared to the NT conditions. Similarly, Hadadi et al. [10] reported that KT notably increased anterior reach distance in the star excursion balance test among individuals with CAI. However, Gehrke et al. [40] found no significant improvement with KT. This discrepancy may be attributed to differences in taping techniques. The peroneal muscle plays a critical role in stabilizing the ankle joint [41]. KT is believed to enhance muscle activation by providing cutaneous stimulation and applying centripetal tension on the fascia [13]. Both our study and Hadadi et al. [10] applied taping to the ankle and calf, including the peroneus longus muscle. In contrast, Gehrke et al. [40] mainly focused on wrapping the ankle joint. They did not specifically target the peroneus longus muscle, which may have limited any improvements in ankle joint stability. Furthermore, Yu et al. [42] suggested that increasing the length of KT could lead to greater improvements in proprioception, indicating that the coverage area may influence the efficacy of KT. It is possible that the taping effect by Gehrke et al. [40] was insufficient to enhance dynamic postural stability in individuals with CAI due to the limited taping range.

Jiang et al. [43] demonstrated that an increase in knee flexion angle favors a lower COG, and a lower COG means better postural stability. The results of this study revealed that individuals with CAI had lower peak knee flexion angles compared to healthy controls. Similarly, Son et al. [44] found that individuals with CAI exhibit smaller knee flexion angles than their healthy counterparts. According to the kinetic chain theory, sensorimotor deficits in the ankle joint of individuals with CAI may affect proximal joint movements, potentially leading to a reduction in peak knee flexion angle [45]. Additionally, this study demonstrated that KT significantly increased peak knee flexion angle in individuals with CAI. Although the knee joint was not taped in this study, the observed improvement in peak knee flexion angle can be attributed to changes in ankle mechanics. Previous studies have indicated a positive correlation between ankle dorsiflexion and knee flexion angles during landing tasks [46, 47]. An increase in ankle dorsiflexion angle facilitates the knee joint in achieving a greater flexion position during movement [48]. This study also revealed a significant improvement in peak ankle dorsiflexion angle, which may have contributed to the observed increase in peak knee flexion angle.

Limited ankle dorsiflexion range of motion is a significant predictor of the risk of ankle sprains [49]. A reduction in ankle dorsiflexion and eversion angles impairs the ability of the trans-ankle muscle-tendon units to absorb impact forces during eccentric contractions. This leads to increased ankle stiffness and elevated load rates, consequently raising the risk of ankle sprains [50]. The results of this study demonstrated that KT significantly increased peak ankle dorsiflexion and eversion angles. Previous studies indicate that enhancing these angles not only positions the subtalar joint in more aligned and safe configuration but also reduces tensile stress on the lateral ankle ligaments, thereby improving ankle stability [51–53]. Therefore, these findings suggested that the application of KT can help individuals with CAI maintain a safer ankle joint position and facilitate the absorption of impact forces. Ultimately, this can reduce the risk of ankle sprains. This study applied KT to the tibialis anterior and peroneus longus muscles, potentially increasing sensory input from mechanoreceptors in these areas. This enhanced sensory input strengthens the composite signals formed by muscle spindles, which are transmitted to the contractile fibers via motor neurons. This regulation of muscle contraction ultimately leads to increased ankle dorsiflexion and eversion angles [54, 55]. Additionally, KT enhances proprioception of the foot and ankle, allowing participants to more accurately perceive movement direction and joint position. As a result, participants exhibit more audacious movements, achieving a more stable ankle position through increased dorsiflexion and eversion angles [56, 57].

### 4.2 Kinetic characteristics

COP represents the weighted average of all pressures exerted over the contact area with the ground. COP displacement serves as an indicator of the postural control system’s “error signal” to some extent [58]. The findings of this study revealed that individuals with CAI exhibited greater mediolateral COP displacement compared to healthy controls, suggesting a decline in postural stability among those with CAI. According to Zhang et al. [59], proprioceptive disorders in individuals with CAI hinder the timely response of ankle joints to external disturbances during weight-bearing activities. This delay can lead to incorrect foot positioning and ultimately disrupt body stability. Growing evidence indicates that alterations in the central nervous system contribute to postural stability deficits in individuals with CAI. Terada et al. [60] employed diffusion tensor imaging to identify reduced white matter microstructure in individuals with a history of ankle sprains. This reduction in white matter microstructure has been linked to deficits in postural control [61, 62]. Furthermore, Rosen et al. [63] found that individuals with CAI exhibited increased cortical activation in the supplementary motor area during single-leg stance compared to healthy individuals. This finding may suggest alterations in the central mechanisms governing postural control among those with CAI.

Furthermore, the findings of this study demonstrated that KT significantly reduced both mediolateral COP displacement and average velocity. Consistent with our results, Li et al. [23] also reported improvements in mediolateral COP displacement among individuals with CAI, when using KT. Both of these studies involved taping the peroneus longus muscle. Yen et al. [64] highlighted that the tension applied by KT to the lateral aspect of the ankle joint not only provides passive mechanical assistance for eversion, but also offers sensory cues that facilitate active eversion. Increasing ankle eversion movement helps correct abnormal ankle inversion in individuals with CAI, guiding the joint closer to a neutral position and improving stability. This improvement may contribute to the observed reductions in mediolateral COP displacement and average velocity.

McGuine et al. [65] found that higher COG sway scores are associated with an increased incidence of ankle sprains. The findings of this study indicate that KT significantly reduced mediolateral COP displacement, suggesting an improvement in mediolateral sway during the Y-balance anterior task in individuals with CAI. Increases in peak knee flexion and peak ankle dorsiflexion angles contribute to a lower body position during single-leg stance, lowering the COG, thereby minimizing body sway. Additionally, Isabel et al. [66] proposed that ankle taping can enhance the confidence of an individual, sense of stability, and security during dynamic balance tasks. Thus, KT may promote actual postural stability by fostering psychological stability, leading to a reduction in mediolateral COG displacement.

### 4.3 Limitations

However, this study has several limitations. First, the electrodes of the surface electromyography device must be attached to the skin, which could interfere with the effects of KT; therefore, muscle activity monitoring was not conducted in this study. Second, due to the limited sample size, the analysis did not separate male and female participants; however, existing research indicates that gender differences can influence biomechanical characteristics [67].

## 5. Conclusion

KT significantly improved abnormal ankle posture by promoting greater ankle dorsiflexion and eversion angles. Additionally, KT reduced mediolateral COP displacement and average velocity to improve postural stability. These changes collectively decrease the risk of ankle sprains. Future studies could analyze the changes in lateral ankle ligament stress by finite element or multiscale models, thereby validating the preventative effect of KT on recurrent ankle sprains. Overall, these findings support the use of KT as a valuable intervention in rehabilitation programs aimed at preventing recurrent ankle sprains in individuals with CAI.

## Supporting information

S1 Data Raw data. (XLSX)
